# The Aichi Biodiversity Targets: achievements for marine conservation and priorities beyond 2020

**DOI:** 10.7717/peerj.9743

**Published:** 2020-12-21

**Authors:** Hannah Carr, Marina Abas, Loubna Boutahar, Olivia N. Caretti, Wing Yan Chan, Abbie S.A. Chapman, Sarah N. de Mendonça, Abigail Engleman, Filippo Ferrario, Kayelyn R. Simmons, Jana Verdura, Anna Zivian

**Affiliations:** 1 The Joint Nature Conservation Committee, Peterborough, Cambridgeshire, UK; 2 Departamento de Ciencias Marinas y Costeras, Universidad Autónoma de Baja California Sur, La Paz, Baja California Sur, Mexico; 3 BioBio Research Center, BioEcoGen Laboratory, Faculty of Sciences, Mohammed V University in Rabat, Rabat, Morocco; 4 Laboratorío de Biología Marina, Departamento de Zoología, Universidad de Sevilla, Sevilla, Spain; 5 Department of Marine, Earth, & Atmospheric Sciences, North Carolina State University, Raleigh, NC, USA; 6 Australian Institute of Marine Science, Townsville, QLD, Australia; 7 School of BioSciences, University of Melbourne, Melbourne, VIC, Australia; 8 School of Ocean and Earth Science, University of Southampton, Southampton, Hampshire, UK; 9 Centre for Biodiversity and Environment Research, University College London, London, UK; 10 Department of Oceanography, Dalhousie University, Halifax, NS, Canada; 11 Department of Biological Sciences, Florida State University, Tallahassee, FL, USA; 12 Québec-Ocean and Département de Biologie, Université Laval, Québec, QC, Canada; 13 Institut d’Ecologia Aquàtica, Facultat de Ciències, Universitat de Girona, Girona, Spain; 14 Ocean Conservancy, Santa Cruz, CA, USA

**Keywords:** Aichi, Biodiversity, Target, 2020, Marine, Conservation, Priorities

## Abstract

In 2010 the Conference of the Parties (COP) for the Convention on Biological Diversity revised and updated a Strategic Plan for Biodiversity 2011–2020, which included the Aichi Biodiversity Targets. Here a group of early career researchers mentored by senior scientists, convened as part of the 4th World Conference on Marine Biodiversity, reflects on the accomplishments and shortfalls under four of the Aichi Targets considered highly relevant to marine conservation: target 6 (sustainable fisheries), 11 (protection measures), 15 (ecosystem restoration and resilience) and 19 (knowledge, science and technology). We conclude that although progress has been made towards the targets, these have not been fully achieved for the marine environment by the 2020 deadline. The progress made, however, lays the foundations for further work beyond 2020 to work towards the 2050 Vision for Biodiversity. We identify key priorities that must be addressed to better enable marine biodiversity conservation efforts moving forward.

## Introduction

Human well-being depends on protecting and preserving the functioning of oceans and coastal ecosystems. Over three billion people rely on these incredibly biodiverse ecosystems for their livelihoods, and even more depend on the services healthy ocean and coastal ecosystems provide, including food, storm surge protection, and cultural resources. Healthy marine ecosystems are essential for maintaining life on Earth; however, the ocean faces unprecedented anthropogenic threats, reducing its ability to provide crucial benefits to humans and other species that depend on it.

To achieve global sustainability and effective ocean stewardship, the international community has pledged to conserve global marine ecosystems, notably through the United Nations Convention on Biological Diversity (CBD) and the United Nations 2030 Agenda for Sustainable Development (2030 Agenda). In 2010, in Nagoya, Japan, the Parties to the CBD adopted the Strategic Plan for Biodiversity 2011–2020 (Strategic Plan). The Strategic Plan is an overarching framework of 20 aspirational benchmarks—termed the “Aichi Biodiversity Targets”—that countries and stakeholders should meet in order to safeguard biodiversity by 2020. In 2015 UN Member States adopted the Sustainable Development Goals (SDGs) which set the direction for achieving global development while protecting the environment we rely on. These are linked closely to the Aichi Biodiversity Targets and Goal 14, which focuses on the need to conserve the ocean and its resources, is of particular relevance to marine conservation efforts.

In the mid-term assessment of progress towards the implementation of the Strategic Plan, the fourth edition of the Global Biodiversity Outlook (GBO-4, Secretariat of the Convention on Biological Diversity, 2014) concluded that although good progress had been made in some areas, it was unlikely that many of the Aichi Biodiversity Targets would be reached by 2020. The report on SDG 14 progress (United Nations, 2017) underlined that existing policies and treaties are still insufficient to combat the adverse effects of climate change, overexploitation of resources, and pollution.

With 2020 on the horizon, the 4th World Conference on Marine Biodiversity (WCMB) in 2018 in Montreal, Canada, implemented a mentoring program to bring together early career researchers with more senior scientists in working groups during the conference to synthesize the achievements of a decade of work on “Strategies for conservation of marine biodiversity”. One of the working groups focused on critically evaluating the role of conservation paradigms and technology in achieving the Aichi Biodiversity Targets. While recognizing that many targets overlap and that all 20 targets are linked to marine conservation, the group identified the most relevant targets to four core themes of marine conservation: restoration (Aichi target 15), protection (Aichi target 11), monitoring (Aichi target 19), and sustainable use (Aichi target 6) (Table 1). In this review paper, the group discusses both the accomplishments towards and shortfalls of achieving these four targets and suggests priorities for marine biodiversity conservation to be considered in the post-2020 Global Biodiversity Framework (“post-2020 Framework”).

**Table 1:** Reviewed Aichi Biodiversity Targets (SCBD, 2010).

Target	Strategic goal	Description
6	B: Reduce the direct pressures on biodiversity and promote sustainable use	By 2020 all fish and invertebrate stocks and aquatic plants are managed and harvested sustainably, legally and applying ecosystem based approaches, so that overfishing is avoided, recovery plans and measures are in place for all depleted species, fisheries have no significant adverse impacts on threatened species and vulnerable ecosystems and the impacts of fisheries on stocks, species and ecosystems are within safe ecological limits
11	C: Improve the status of biodiversity by safeguarding ecosystems, species and genetic diversity	By 2020, at least 17% of terrestrial and inland water, and 10% of coastal and marine areas, especially areas of particular importance for biodiversity and ecosystem services, are conserved through effectively and equitably managed, ecologically representative and well-connected systems of protected areas and other effective area-based conservation measures, and integrated into the wider landscape and seascapes
15	D: Enhance the benefits to all from biodiversity and ecosystem services	By 2020, ecosystem resilience and the contribution of biodiversity to carbon stocks has been enhanced, through conservation and restoration, including restoration of at least 15% of degraded ecosystems, thereby contributing to climate change mitigation and adaptation and to combating desertification
19	E: Enhance implementation through participatory planning, knowledge management and capacity building	By 2020, knowledge, the science base and technologies relating to biodiversity, its values, functioning, status and trends, and the consequences of its loss, are improved, widely shared and transferred, and applied

As the CBD contracting parties continue discussing how to meet the 2050 Vision for Biodiversity (CBD, 2020), numerous resulting assessments stress the need to build off the past decade’s achievements by critically evaluating implementation of the Strategic Plan and demonstrating concrete examples of success. Consultation groups also emphasize need for including input from diverse perspectives, including youth, in the post-2020 Framework (CBD, 2019a, 2019b). Our synthesis of progress made towards Aichi targets 6, 11, 15, and 19, and respective future priorities, can serve as guidance for future CBD Working Group discussions.

## Review

Aichi target 6—By 2020 all fish and invertebrate stocks and aquatic plants are managed and harvested sustainably, legally and applying ecosystem based approaches, so that overfishing is avoided, recovery plans and measures are in place for all depleted species, fisheries have no significant adverse impacts on threatened species and vulnerable ecosystems and the impacts of fisheries on stocks, species and ecosystems are within safe ecological limits.

There have been some successes over the last decade against Aichi target 6.

The number of stocks fished within biologically sustainable levels has increased in the Northeast Atlantic, Australia, and broadly across the Pacific. However, the general trend for global fisheries remains negative as the percentage of stocks fished at biologically unsustainable levels increases (FAO, 2018). Challenges to achieving sustainable fisheries include insufficient data, habitat loss, and a disconnect between humans and the environment (Hazen et al., 2016). Spatial constraints and jurisdictional boundaries can also pose challenges to taking ecosystem-based management (EBM) approaches to fisheries and requires spatial management units that recognize both local ecological and socio-economic benefits (Sanchirico & Wilen, 2005; Kenny et al., 2018). Implementing a holistic ecosystem-based approach to management is complex, especially because views vary on the fundamental principles of such an approach (Long, Charles & Stephenson, 2015).

Different considerations for developing vs developed countries can also make governance challenging; for example engaging multiple stakeholders, adopting decentralized governance strategies, and achieving long term sustainability through EBM approaches can be easier in wealthy developed countries with greater resources (Gutierrez, Hilborn & Defeo, 2011; DuBois & Zografos, 2012; Cinner et al., 2016; Kenny et al., 2018; Teh et al., 2018). In addition, fishing access agreements with wealthy countries permitting the harvesting of demersal and highly migratory species within the Exclusive Economic Zones (EEZ) of developing countries (Gagern & Van den, 2013) can create a challenge for sustainable fisheries within these developing countries’ waters.

At the local level, sustainable management of smaller artisanal fisheries can be realized through place-based strategies and an increased awareness of socio-economic and cultural benefits such as enhanced food security (Kittinger et al., 2015; Stuart et al., 2019). Communities with strong cultural connections to coastal resources are, however often data limited and lack resources for combating the knowledge gap in applying market-based approaches and industrial supply chains to small-scale fisheries (Smith, 2008; O’Rourke, 2014; Kittinger et al., 2015).

Although many preventative actions have been implemented at various scales to address overfishing and depleted stocks (European Union, 2008; Papaioannou, 2016; FAO, 2018; Oozeki et al., 2018; Satria et al., 2018), illegal, unreported, and unregulated (IUU) fishing remains a threat globally for marine conservation efforts. Improved technological, enforcement, and/or compliance measures are needed to tackle this challenge across and beyond national jurisdictions.

While some regional fish stocks have shown recovery (OSPAR Commission, 2017; Van Gemert & Andersen, 2018) through improved management, many are still in decline. Climate change poses a growing challenge to developing and implementing recovery plans in addition to predicting optimum sustainable yield (Vert-pre et al., 2013; Britten et al., 2017; Teh et al., 2018). As ocean temperatures continue to rise, low latitude or warm-water species are projected to migrate poleward (Jones & Cheung, 2015; Morley et al., 2018) leading to increased catch at higher latitudes and decreased catch in lower latitudes (Cheung, Watson & Pauly, 2013; Cheung et al., 2016; Blanchard et al., 2012). Changes in marine fish stock distribution occur on local and regional scales, with decreasing harvests predicted in the tropics and regions nutritionally and economically dependent on fisheries (Bell et al., 2016; Barange et al., 2014). For all fisheries, EBM, including science-based recovery plans, stakeholder agreements, and human behavioral change, are critical for sustainable harvesting of fish, shellfish and aquatic plants within safe ecological limits for future generations.

Greater success in meeting the aims of Aichi Target 6 can be achieved through identifying priority areas of focus and attainable outcomes for sustainable, EBM using long-term adaptive approaches (Teh et al., 2018) which consider climate change. For instance, recent reviews on shark and ray conservation have illustrated the knowledge gap in the coupled dynamics of human dimensions (e.g., socio-economic status, political stakeholders, market demands, enforcement capacity) and natural population drivers (e.g., life history traits, habitat availability) (Gaymer et al., 2014; Dent & Clarke, 2015; Jaiteh, Loneragan & Warren, 2017; Martin, Maris & Simberloff, 2016; Martins et al., 2018), which could be targeted as a priority area for research. Adaptive conservation solutions and policies should be adopted in the face of global disparities, such as governance, resource dependance, and monitoring and enforcement capacity (Lucifora et al., 2019; Mizrahi et al., 2019) that often stifle progress.

The post-2020 Framework should consider a few priority areas. Recovery plans and avoidance of overfishing of stocks should focus on flexible solutions at different scales that target knowledge gaps and the address the challenges posed by the highly mobile, transboundary nature of many fish stocks. The negative impacts environmental impacts of fishing needs to continue to be addressed with a focus on adverse impacts on vulnerable ecosystems and threatened species, along with IUU. Cross-boundary, governmental, and institutional sharing of data and best management practices should be improved to help develop a more transparent, adaptive, resilient, and robust framework for sustainable EBM fishing practices.

Effective implementation of recovery plans and ending overexploitation of current stocks require a greater understanding of the parallel components of species life history and gear-related mortality. Life history traits often explain demographic characteristics that drive stock dynamics, such as size at maturity, age of recruitment, and maximum sustainable yield. These characteristics are even more important in data-poor habitats such as the deep sea, where organisms are slow growing, long living, and often have lower reproduction potential, making these ecosystems highly vulnerable to overfishing (Koslow, 1997; FAO, 2009). Long-term, flexible management planning for highly vulnerable ecosystems should therefore include realistic timespans to distinguish natural fluctuations in population dynamics from impacts of overfishing (Clark et al., 2016). Incorporating knowledge of fish physiology, spatial behavior of stocks, gear interactions, and economic values into stock assessments at the global scale can also improve recovery efforts (Lynch, Shertzer & Latour, 2012; Poloczanska et al., 2013; Lagasse et al., 2015; Horodysky et al., 2016). Managers should therefore develop flexible and adaptive recovery plans that incorporate information on life history traits and population dynamics, measures for stochastic biological and environmental events (Pershing et al., 2015; Britten et al., 2017), as well as strategic spatial plans for monitoring and enforcement (Costa et al., 2018). Implementing these strategies will allow policy makers to address changes in global fisheries as a result of climate-driven stressors. Target-based spatial planning software such as Marxan (Ball, Possingham & Watts, 2009) or Zonation (Moilanen, 2007) can facilitate an adaptative framework, allowing resource managers to use an array of biological information along with fishing effort data to improve time-space management approaches in addition to addressing economic and political insecurities for developing nations (Kathijotes, 2013) or nations at risk from climate-induced stressors (Mercer et al., 2012; Mamauag et al., 2013).

Current efforts to reduce negative fisheries impacts on a global scale include approaches such as bycatch reduction strategies (Swimmer et al., 2011; O’Neill & Mutch, 2017; Veiga-Malta et al., 2018), bans on harvests of certain species (Sherman et al., 2018), elimination of discards (European Union, 2013), regulating exports related to non-sustainable fisheries (Shiffman & Hueter, 2017), fishing regulatory closures for Vulnerable Marine Ecosystems (VME) in Areas Beyond National Jurisdiction (ABNJ) (NEAFC, 2018), prohibition of destructive gear types for VMEs (Auster et al., 2011), marine protected areas (Hameed et al., 2017; Hastings, Gaines & Costello, 2017), and increased aquaculture production (FAO, 2018; Froehlich, Gentry & Halpern, 2017). Implementation of conservation measures such as large-scale marine protected areas can pose challenges for nations that close off areas of their waters; however cross-jurisdictional management with transferable fishing rights has been proposed as a potentially viable market-based solution to promote transboundary cooperation in these situations (Villaseñor-Derbez, Lynham & Costello, 2020). Major concern remains, however, for sustainable management of fish stocks in ABNJ (Cullis-Suzuki & Pauly, 2010) and about the impacts of fishing activities to deep-sea ecosystems (Koslow et al., 2000; FAO, 2008; Brock et al., 2009; Ramirez-Llodra et al., 2011), as well as IUU fishing in polar areas (Ainley & Pauly, 2014; Österblom, Bodin & Rashid Sumaila, 2014; Nyman, 2018).

Understanding adverse impacts from overfishing should include historical, present, and projected priority areas to inform better implementation of global ecosystem-based practices. Future practices for regions that have multiple fisheries and/or rely on potentially harmful gear types (e.g., bottom trawls, bottom longline) (Clark & Koslow, 2008; Halpern et al., 2007) should be critically evaluated at an ecosystem level. As deep-sea fisheries become more prevalent because of recent advances in gear technology, precautionary measures should be put in place to assess damage and recovery potential. Long term impacts of gear interactions in the deep sea can have delayed responses reflected in a degraded seabed environment and remaining fauna (Clark et al., 2010; Williams et al., 2010). A feedback mechanism for management that includes historical data and periodically updated models or rapid assessments would provide a process for extensive review and integration of new information.

The sharing of management outcomes, information on species life history across regions (e.g., ontogenetic shifts, foraging behavior), and guidelines and best practices across boundaries (disciplinary, political, governmental, institutional, sectoral) could be improved through the use of information sharing frameworks. For example, regional guidelines and approved practices for global issues such as invasive species and highly migratory fish stocks have been successful in coordinating common language and strategies across political and regional boundaries (Lascelles et al., 2014; Morris, 2012; Serdy, 2016). Governance of fish stocks between communities at local scales would also benefit from sharing resources (e.g., biological sampling, analytical modeling), use of traditional ecological knowledge, and active engagement of the public (Tallman, Roux & Martin, 2019). Open communication and flexible solutions may allow for better decision-making and cooperation among stakeholders that differ in socio-economic status. Cross-boundary, governmental, and institutional sharing of best management practices will also enhance the capacity to holistically evaluate the efficacy of current recovery plans and fisheries mitigation impacts. The use of a more robust, collaborative framework, inclusive of climate change impacts, population drivers, and socio-economic information across regions will aid in assessing how to achieve sustainable harvest of fish stocks within safe ecological limits for all marine fauna and their living environment.

Aichi target 11—By 2020, at least 17% of terrestrial and inland water, and 10% of coastal and marine areas, especially areas of particular importance for biodiversity and ecosystem services, are conserved through effectively and equitably managed, ecologically representative and well-connected systems of protected areas and other effective area-based conservation measures, and integrated into the wider landscape and seascapes.

As a target obligating Contracting Parties to meet measurable commitments by 2020 (Campbell et al., 2014), and as the most intensively studied of the Aichi targets (Green et al., 2019), Target 11 should have proven a relatively easy target to assess in terms of achievements for marine conservation and associated future priorities. However, work towards this target to date appears to have had complex, and sometimes conflicting, outcomes. For instance, progress towards area-based protection seems to have resulted in less attention being paid to the management, representativeness, broader integration, and connectivity of conservation measures (i.e., qualitative measures), which are also specified components of Target 11.

Progress towards the quantitative part of the target—for 10% of coastal and marine areas to be protected (Convention on Biological Diversity, 2011)—has been much slower than progress towards the terrestrial equivalent, which aims for protection of at least 17% of inland areas (Watson et al., 2014). The total ocean area covered by marine protected areas (MPAs) increased from 2.3% in 2011 (Leadley et al., 2014) to 7.91% in 2020 (UNEP-WCMC & IUCN, 2020a). However, protected area coverage is uneven, with most MPAs restricted to national waters, covering around 18.45% of exclusive economic zones (EEZ), and only 1.18% of Areas Beyond National Jurisdiction (ABNJ; UNEP-WCMC & IUCN, 2020a). To be ecologically representative, MPAs need to protect the full range of habitats found within our ocean, both nearshore and away from the coast. There is also socioeconomic disparity in achieving this target. For instance, while 40% of high-income countries already exceed the 10% minimum for protected national marine surface area, 75% of the waters of developing nations protect less than 2% (Failler, Touron-Gardic & Traore, 2019). Further work still needs to be undertaken to understand the contribution that other effective area-based conservation measures could make to the achievement of the target (ICUN WCPA, 2018).

Furthermore, it has been argued that a recent focus on area-based measures of progress towards Aichi targets has not resulted in the protection of a diverse range of habitats (Fischer et al., 2019) nor of the most threatened areas (Kuempel et al., 2019). For instance, despite the contributions of ABNJ to marine biodiversity and ocean health (Barbier et al., 2014), and the relatively higher vulnerability of life in this area (O’Hara et al., 2019), high seas habitats remain underrepresented in current protected areas. Steps have been made towards conserving deep-sea ecosystems via various protected-area-oriented policies and projects (Calado et al., 2011; DFO, 2017; OSPAR Commission, 2017), but most of the active hydrothermal vent environments that have been protected to date fall within country jurisdictions across the globe (Menini & Van Dover, 2019; Van Dover et al., 2012) and spreading ridges are particularly underrepresented in MPAs globally (Fischer et al., 2019).

The recent acceleration towards the establishment of very large MPAs (Boonzaier & Pauly, 2016), such as the Ross Sea Protected Area and the Papahānaumokuākea Marine National Monument, has resulted in a rapid increase in percentage of global oceans within MPAs. An analysis in 2014 found that ten MPAs (existing or under creation) accounted for more than 53% of the world’s total MPA coverage in 2014 (Devillers et al., 2015; Watson et al., 2014); since then many others have been created (Marine Conservation Institute (MCI), 2020). Therefore, without these large MPAs, the 10% target would be even further from being reached. Moreover, MPAs within Large Marine Ecosystems do not represent the majority of geomorphic features and habitats found within them (Fischer et al., 2019).

Although some progress is being made towards the quantitative element of the target, it is widely recognized that further work is required on its qualitative components (Campbell & Gray, 2019; Green et al., 2019; Lemieux et al., 2019). Carr et al. (2017) elaborate on the importance of various types of ecological spatial connectivity—population, genetic, community, and ecosystem—that refer to the movement of genes, individuals, species, nutrients, and materials between areas. Considering biodiversity representation and connectivity within MPA networks can enhance biodiversity persistence and efficiency of conservation (Magris et al., 2018). Thus, design and implementation strategies have changed from designating single, unrepresentative MPAs to establishing large and inclusive MPAs and important networks of MPAs, such as on the California coast and in the Northeast Atlantic (OSPAR, 2006; California Department of Fish and Wildlife, 2016). Despite increasing efforts to create MPA networks and improve ecological connectivity, few MPAs (11% of 746 MPAs) explicitly mentioned connectivity as an ecological criterion as of 2019 (Balbar & Metaxas, 2019). In particular, there is a real paucity of research on larval ecology and dispersal in VMEs, such as deep-sea habitats (Hilário et al., 2015), where species are strongly adapted to specific conditions. For example, chemosynthetic habitats are so distinct that so-called “stepping-stone environments” might be far apart from one another, limiting connectivity among distant MPAs and requiring protection spanning large “metacommunities” (Mullineaux et al., 2018; Suzuki et al., 2018). While the importance of connectivity in conservation planning strategies is still under debate (Costello & Connor, 2019), this concept should still be considered in MPA planning for all ecosystems, especially in cases over longer distances (Manel, Loiseau & Puebla, 2019) and should increasingly use genetic data sources (Xuereb et al., 2019; Zeng et al., 2019).

Information on MPA management measures is lacking globally, and the effectiveness of these measures at maintaining or restoring biodiversity is still under great debate (EEA, 2015; MedPAN & UNEP-MAP-SPA/RAC, 2016; Rees et al., 2018; Sala et al., 2018; Campbell & Gray, 2019). Currently only 1% (UNEP-WCMC & IUCN, 2020b) to 4.8% of global MPAs are considered fully implemented and managed (Marine Conservation Institute (MCI), 2020). The rest of the designated MPAs either have not implemented or evaluated existing management strategies (UNEP-WCMC & IUCN, 2020b) or still allow significant extractive activities (Giakoumi et al., 2017). For coral reefs, 47% of MPAs were ineffective, 38% partially effective, and only 15% fully effective (Burke et al., 2011). In this case, the lack of effectiveness was a result of the management framework being ignored or not enforced; insufficiently addressing threats within borders of MPAs via regulation; and the location of MPAs failing to address real threats to reefs (Burke et al., 2011). Meanwhile, Lyme Bay MPA in the UK provides a contrasting example of the benefits of addressing qualitative measures, where involving stakeholders in conservation decisions facilitated ecological recovery and improvements to equity amongst different fishing groups (Johnson et al., 2019). This is also done within the MPA network in California, where a commitment to monitoring, coordination, public education, and enforcement, along with strong public and stakeholder engagement, has led to demonstrated success (Murray & Hee, 2019). Overall, beyond size, these studies emphasize how the equitable management of MPAs shapes conservation effectiveness.

The qualitative components of Target 11 are also affected by complex socio-economic and environmental trade-offs (Zafra-Calvo et al., 2019). On larger scales, the implementation of coastal or large MPAs in developed and developing countries is hampered by economic forces (Amengual & Alvarez-Berastegui, 2018; Driscoll et al., 2018), which can lead to instances where the government proposes the removal of protections, for example against mining (Pinheiro et al., 2019). In high-income countries of the Mediterranean, most long-term MPA monitoring programs have been carried out with the support of research projects and were only occasionally financed by the MPA management body (Rilov et al., 2019). By contrast, in developing countries, economic development remains the main public policy concern, which is insufficiently geared towards environmental protection (World Bank, 2017). Thus, we suggest that a future research priority should involve the investigation of the values humans place on different aspects of biodiversity and ecosystem functioning, as well as improved communication on the benefits provided by MPAs, as there have been cases illustrating the potential improvements to MPA effectiveness facilitated by incorporating social outcomes in MPA design (MacKeracher, Diedrich & Simpfendorfer, 2018; Folkersen, Fleming & Hasan, 2019).

Looking to the future, we propose that experiences, data, and guidelines should be shared across political boundaries. Technological advancements and accumulated baseline data should improve identification of ecosystems in critical need of protection, while international communication among managers and researchers can facilitate global information exchange, leading to the early identification and protection of at-risk ecosystems. Building a platform for this knowledge exchange should be a key priority of the post-2020 Framework and could build on existing data-sharing platforms (such as Ocean Biogeographic Information System (www.obis.org) or Global Biodiversity Information Facility (www.gbif.org), and will be necessary to prevent confusing contradictions (Gownaris et al., 2019) and to ensure conservation strategies can be effective in both national and international waters, across a variety of habitats.

The effective implementation of conservation strategies is a crucial part of achieving conservation success. Proper implementation requires identifying the most effective conservation strategies, optimizing regulations to promote sustainable use of marine resources, involving local dependent communities, and using more efficient and effective monitoring and enforcement methods (Lemieux et al., 2019). Identifying the best methods for each component will require collaboration among multiple stakeholders, as well as the appropriate resources to implement these strategies, which has proved challenging thus far. Since the ocean is an interconnected and dynamic system, there is a critical need to ensure effective management of all ocean areas and uses. Thus, we propose that less well-known areas, such as deep-sea ecosystems and ecosystems in countries with limited financial and technological capacity, should be prioritized for data collection and capacity-building within the post-2020 Framework, as comprehensive datasets are required to minimize bias in protected-area representation (Roberts, Duffy & Cook, 2019). This will require collaboration between scientists, managers, civil associations, and policymakers (Juniper et al., 2019). Novel approaches for more efficient data collection, dissemination, and re-use should also be an urgent priority (Edgar et al., 2016), as well as the incorporation of volunteer-contributed data and local and traditional knowledge into conservation decision making. New metrics may be required to incorporate data from such a variety of sources and to assess the effectiveness of conservation measures (Jantke et al., 2018; McQuatters-Gollop et al., 2019).

Finally, even within effectively managed MPAs, another great challenge remains for marine conservation—climate change (Bruno et al., 2018). Already, elevated temperatures have led to the collapse of coral reefs, which are some of the most biodiverse ecosystems in tropical regions (Hughes et al., 2017). Expanding our understanding of climate-driven changes to the ocean is a key priority to better comprehend and anticipate their impacts on marine biodiversity. Appropriate baseline data is fundamental to this, and past and present data must be used to model future scenarios (Nicholson et al., 2019). Incorporation of these data and models into coastal and ocean planning is essential (Legg & Nagy, 2006; Nicholson et al., 2019) within MPA network design guidelines (Munguia-Vega et al., 2018), or in protection and restoration plans for habitats vital for carbon sequestration and storage. Protection from anthropogenic stressors through MPAs may improve the resilience of fauna ahead of climate change impacts (Bates et al., 2014). Species sensitive to ocean warming, however, are not guaranteed protection even by the most effectively managed MPA. For example, due to the warming of coastal waters, sea urchin populations have totally disappeared from a marine reserve in the southeastern Mediterranean that has been protected for more than two decades (Rilov, 2016). Additionally, species ranges may change as a result of climate change impacts, moving populations outside of MPA boundaries. As such, a flexible, adaptive approach needs to be taken that allows for boundary modifications of MPAs in response to these changes.

Recent developments in discussions informing the post-2020 Framework have called for the 10% quantitative element of this target to increase to 30% (CBD, 2020). Whether we consider the Aichi Target of 10%, or the ambitious new 30% target, the result is the same: we still have far to go as a global community to ensure that protected areas adequately conserve the ocean and its valuable resources.

Aichi target 15—By 2020, ecosystem resilience and the contribution of biodiversity to carbon stocks has been enhanced, through conservation and restoration, including restoration of at least 15% of degraded ecosystems, thereby contributing to climate change mitigation and adaptation and to combating desertification.

Anthropogenic impacts have eroded the resilience of many ecosystems across the globe, triggering significant declines and losses of many of them (UNEP, 2006), resulting in a reduction of the services they provide, and negatively impacting human well-being. In order to improve ecosystem resilience, there is increasing interest in not just conservation but also restoration. In this section, we review six highly valuable global marine and coastal habitats to illustrate the services they provide (Table 2), their status, and the efforts to restore them.

**Table 2:** Examples of the ecosystem services provided by coral reef, mangrove, seagrass, seaweed forest, saltmarsh, and oyster reef habitats.

Habitat	Services
Coral reefs	Coastal protection (Ferrario et al., 2014; Spalding et al., 2014); climate change mitigation (Spalding et al., 2014); food provision (Teh, Teh & Sumaila, 2013); nutrient cycling; primary productivity; habitat provision (McWilliam et al., 2018); support of high biodiversity (Bellwood, Hoey & Choat, 2003; Fisher et al., 2015).
Mangroves	Carbon sequestration (Duarte, Middelburg & Caraco, 2005; Gress et al., 2017); coastal protection and climate change mitigation (Spalding et al., 2014); habitat provision (Nagelkerken et al., 2008; Kimirei et al., 2013).
Seaweed forests	Primary production; nutrient cycling; habitat provision; food provision; coastal defense (Dayton, 1985; Steneck et al., 2002; Smale et al., 2013); and potentially carbon sequestration (Krause-Jensen & Duarte, 2016).
Seagrass beds	Carbon sequestration (Duarte, Middelburg & Caraco, 2005; Fourqurean et al., 2012); coastal protection (Fonseca & Cahalan, 1992); primary productivity; food provision; habitat provision; support of high biodiversity (Duarte & Chiscano, 1999).
Saltmarshes	Carbon sequestration (Chmura et al., 2003; Duarte, Middelburg & Caraco, 2005; Drake et al., 2015); coastal protection (Möller et al., 1999; Shepard, Crain & Beck, 2011; Spalding et al., 2014); climate change mitigation (Spalding et al., 2014); habitat provision (Barbier et al., 2011).
Oyster reefs	Coastal protection and sediment stabilization (Rodriguez et al., 2014; La Peyre et al., 2015); nutrient cycling and water filtration (Zu Ermgassen et al., 2012; Kellogg et al., 2014); food and habitat provision (Peterson, Grabowski & Powers, 2003; Grabowski et al., 2012; Gilby et al., 2018); commercial and recreational harvest (NMFS, 2017).

Coral reefs have declined worldwide, with 50% of Great Barrier Reef corals dying in 2016 and 2017 alone (Hughes et al., 2019). Reef restoration has been attempted via culturing of asexually produced coral fragments and occasionally the rearing and settling of coral larvae (Guest et al., 2014); however, coral restoration is still in its infancy. Some small-scale successes have occurred in recent years (Guest et al., 2014; Cruz & Harrison, 2017); for example, a breeding population of one coral species (i.e., *Acropora tenuis*) was re-established in 3 years by deploying coral larvae into mesh enclosures in the reefs (Cruz & Harrison, 2017).

Similarly, nearly 50% of the world’s mangrove forests have disappeared since the 1960s (Giri et al., 2011). Restoration attempts have been made, but at a smaller scale and success rate than depletion has occurred (Romañach et al., 2018). For example, half of the 67 planting efforts in Sri Lanka since 2004 showed no surviving plants; only 200–220 hectares have been successfully restored (Kodikara et al., 2017).

Despite the fact that global declines of seaweed forests are small on average, significant declines have been confirmed at the local scale in 38% of ecoregions (Krumhansl et al., 2016). Although seaweed restoration is gaining recognition (Gianni et al., 2013), there have only been a few long-term (Verdura et al., 2018) and large-scale successful restoration projects (Campbell et al., 2014). Seagrass beds are also declining, and almost 28% of their global extent has been lost since 1879 (Orth et al., 2006; Waycott et al., 2009). Since the 1970s, seagrass restoration trials have rapidly increased worldwide (Paling et al., 2009; Van Katwijk et al., 2016), but only a few successful projects have been reported, mainly due to low performance rates and small-scale efforts of the restoration actions (Bayraktarov et al., 2016; Van Katwijk et al., 2016). Similarly, saltmarshes are severely threatened; approximately 50% of salt marshes worldwide have been lost or degraded due to environmental stress and human disturbances (Adam, 2002; Valiela et al., 2009). In denuded areas, natural regeneration might be difficult (Laegdsgaard, 2002), and despite salt marsh restoration’s receiving more attention and having higher success rates than other marine coastal habitats, large-scale restoration projects (of more than a few hectares) are still needed (Bayraktarov et al., 2016).

Substantial historical and continued extraction of oysters via fishing has led to >85% loss in oyster reefs worldwide and declines in oysters due to overharvest have been exacerbated by degraded water and habitat quality (Beck et al., 2011; Diggles, 2013; Peters et al., 2017). Several small- and medium-scale restoration efforts have taken place in response to this decline with mixed success (Schulte, Burke & Lipcius, 2009; Powers et al., 2009; Beck et al., 2011; Puckett & Eggleston, 2012; La Peyre et al., 2014; Hernandez et al., 2018); most of these restoration efforts were in the US, even though oyster reefs occur in both temperate and tropical locations globally. Aquaculture of native oyster species has also become a focus, providing oysters for harvest and delivering additional ecosystem services like those provided via restoration, but the benefits and extent to which aquaculture provides these additional ecosystem services has not yet been thoroughly reviewed (Higgins, Stephenson & Brown, 2011; Alleway et al., 2019).

Restoring and managing these crucial habitats will continue to be a challenge (La Peyre et al., 2014), especially due to shifting baselines because of climate change (Lemasson et al., 2017; Van Oppen et al., 2017; Wood et al., 2019). Of the 62 countries assessed in 2016, only 50% had set national goals with clear alignment with Target 15, and fewer had set quantitative targets (Secretariat of the Convention on Biological Diversity, 2019). Despite many countries taking positive steps, efforts have not been enough to reach the 15% target and further development of methods and techniques for successful restoration is essential for the post-2020 Framework. This includes developing and using indicators of ecosystem degradation and restoration, such as water quality, carbon stocks, fish stocks, and species diversity (Secretariat of the Convention on Biological Diversity, 2019).

Generally, the failure of restoration plans is mainly linked to low survival rates (<52% mean survival during the first 2 years post restoration) and to the small scale (a few hectares or less) that restoration plans normally span (Bayraktarov et al., 2016). Moreover, filling the knowledge gaps that have limited restoration of these ecosystems on a global scale to date should be prioritized. For instance, acquiring and synthesizing information on the historical and present distribution, species-specific population demographics, habitat requirements (Bayraktarov et al., 2016; Hernandez et al., 2018; Puckett et al., 2018; Theuerkauf, Eggleston & Puckett, 2019), and habitat pre-degradation state (Secretariat of the Convention on Biological Diversity, 2019) will be essential. Additionally, restoration success could be enhanced by better understanding how the spatially explicit distribution and heterogeneity of habitats can affect the occurrence and strength of between-species interactions, habitat connectivity, or favorable environmental conditions (Boström et al., 2011). The establishment of systematic monitoring and habitat mapping initiatives will be crucial in supporting restoration through the adoption of a seascape or context-appropriate approach at multiple scales.

Due to the important role the habitats discussed here can play in maintenance of biodiversity, climate regulation, and climate change adaptation and mitigation, further efforts to restore these habitats will be vital (IPCC, 2018). To preserve marine biodiversity and promote ecosystem resilience to mitigate or adapt to future threats, restoration should be linked to the increasing understanding of the response of marine ecosystems to climate change and its interaction with other stressors. In this context, we suggest that global information exchange of baseline data together with technologically advanced tools (e.g., modeling) will be key for managers and researchers to identify best practices for ecosystem restoration and conservation. This also includes understanding past and present data, the natural/unimpacted state of the ecosystem prior to perturbations, and responses of ecosystems to different threats based on experimental and observational data, and on modeling and prediction. Such knowledge will allow managers and researchers to identify the most suitable areas for restoration or conservation. Specific examples include areas with less severe predicted climate change scenarios; already affected areas where restoration could mitigate future threats; or areas of special interest, such as those harboring high local or regional diversity and/or valuable ecosystems.

Aichi target 19—By 2020, knowledge, the science base and technologies relating to biodiversity, its values, functioning, status and trends, and the consequences of its loss, are improved, widely shared and transferred, and applied.

Scientific knowledge of biodiversity and ecosystem functioning has significantly increased in the past decade through increased capacity, implementation, and awareness of the benefits of monitoring at a higher spatial and temporal resolution. The development and increasingly widespread use of innovative technologies and informatics has allowed for non-invasive and low-cost methods for long-term, continuous measurements of biodiversity. These technologies include automated monitoring systems such as passive and active acoustic monitoring (Buscaino et al., 2016; McWilliam et al., 2017; Monczak et al., 2017; Egerton et al., 2018), autonomous underwater vehicles (Griffin et al., 2017; Ferrari et al., 2016, 2018), and high-resolution models of critical habitats for biodiversity (e.g., Allen Coral Atlas https://allencoralatlas.org/, structure-from-motion and benthic imagery models) (Asaad et al., 2019; Friedman et al., 2012; Burns et al., 2015; Ismail, Huvenne & Masson, 2015; González-Rivero et al., 2017).

The establishment of expansive monitoring network programs has enabled greater coverage for biodiversity monitoring and a platform for data sharing among scientists at local (LTER Network, 2018), regional (SponGES, 2018), and even global scales (e.g., OBIS, Global Ocean Observing System (GOOS), MarineGEO (https://marinegeo.si.edu/)) (Saeedi et al., 2019). The increasing use of citizen science, through app-based programs such as SeagrassSpotter (https://seagrassspotter.org/) and iNaturalist (https://www.inaturalist.org/), has also facilitated quick and widespread data collection and processing, which are crucial for rapid biodiversity assessments (Dickinson et al., 2012; Marshall, Kleine & Dean, 2012; Zenetos et al., 2013). Long-term citizen science projects can also document ecosystem change, which is especially important for data-poor regions with limited resources, such as the coral reef systems in Indonesia (Gouraguine et al., 2019).

Despite dramatic increases in data collection over the past decade, these resources have yet to be fully extended to and applied in marine biodiversity policy and management (Heck, Dearden & McDonald, 2012; Petes et al., 2014). Translating data into conservation requires that data repositories contain high quality and widely accessible biodiversity resources, which can inform implementation of EBM. Additional research must be done to understand the effectiveness of big, unstructured datasets associated with mass sampling and citizen science (Bayraktarov et al., 2019) to address biodiversity questions.

Increased efforts to standardize the collection of relevant, targeted biological data (e.g., Essential Ocean Variables and Essential Biological Variables), sampling methods, and paired environmental and metadata has already enabled better data sharing among scientific groups and between scientists and managers, which can promote more rapid and efficient legislative and management action (De Pooter et al., 2017; Muller-Karger et al., 2018; Klein et al., 2019). Protocols aimed at shifting current benefit-sharing and accessibility paradigms among governments, stakeholders, and scientists would also ensure resource databases better serve greater societal needs such as interdisciplinary knowledge exchange, scientific capacity-building, and resource equity. Future guidelines should highlight the increased societal value of databases that remove barriers to sharing and accessing resources (i.e., that are user-friendly and openly accessible). Such guidelines could elicit use of indigenous knowledge and stakeholder engagement, especially at a local scale, which are an important part of improved conservation outcomes (e.g., in MPA design) (Saarman et al., 2013; Burt et al., 2014).

As technology continues to rapidly outpace policy reform, the post-2020 Framework must also address protocols for handling emerging issues with data collection, sharing, and application. Specific guidelines should address marine genetic resources and their impact on biodiversity and conservation going forward. Such guidelines should thoroughly outline protocols for current and future scientific and private-sector acquisition and distribution of genetic resources, including how to address future technological advancements surrounding biodiversity genetics. Although many nations are still developing policy around genetic resources—and/or participating in the Nagoya Protocol on Access and Benefit Sharing—this international framework should communicate benefits and responsibilities of resource sharing among stakeholders, governments, and scientific communities (Harden-Davies & Gjerde, 2019). The biodiversity framework should work in conjunction with policy resulting from early 2020 Nagoya Protocol meetings to establish applicable and actionable guidelines for access and benefit sharing as it pertains to marine biodiversity resources.

Incomplete application of science-informed policy means that despite advances in knowledge, data, and technology, this target has only been partially achieved in the marine environment. The post-2020 Framework should prioritize research on the efficiency of unstructured data, citizen science, and other tools or strategies to inform implementation of EBM. Increasing societal participation in and awareness of biodiversity conservation should also be prioritized, in order to address outstanding knowledge gaps and contribute to an integrated solution for applying science to marine policy and management (Hyder et al., 2015; Jarvis et al., 2015; Theobald et al., 2015). Priority should also be given to developing a framework for cross-boundary resource and benefit sharing as it relates to current biodiversity data and future resources arising from technology and genetic data advancements. Prioritizing these three areas will maximize the impact and longevity of guidelines going forward. Specifically, new legislation regarding biodiversity targets should be created within an adaptive framework that relies on frequent reporting and review of existing policy, both globally and within contracting party countries. This adaptive framework will require discussion among multiple stakeholders and institutions that monitor biodiversity status and will encourage cross-boundary cooperation among groups by holding them accountable for reliable and up-to-date reporting. It will also provide opportunities for the incorporation of emerging data and technologies into policy, and for flexibility of policy in response to unstable conditions driven by climate change. Increased reliance on globally accessible databases and data management frameworks, such as OBIS, by scientists and managers will be required (Klein et al., 2019). Clear metrics for policy evaluation should also be outlined to streamline efficient reporting and review and encourage a common language across sectors. At the global level, successful strategies for EBM should be shared to provide examples for regions struggling with effective and sustainable management; at the local level, positive incentives should be created for EBM to engage multiple stakeholders and partnerships among local communities and managers.

Incorporating a human-perspective approach will be key to addressing biodiversity challenges related to applying science-informed policy moving forward. Mainstreaming biodiversity in policy and the private sector will promote further sustainable development. Linking the post-2020 Framework to other societal challenges such as climate change, human health, and disaster risk reduction, will better connect biodiversity to goals of other goals and commitments, in turn strengthening the use of common language and information sharing across boundaries. Mainstreaming biodiversity in communications and encouraging ocean literacy will help individuals see value in nature and encourage involvement in nature-related decisions that may directly affect them and their communities. One approach to this is incorporating a nature-based curriculum in schools (Lawson et al., 2019). Clear communication of the benefits provided by, and cost of inaction towards, biodiversity targets in terms of ecological, social, economic, and political stability is necessary to increase societal awareness of these issues. Providing tangible alternatives that integrate sustainable actions with social benefits will also be important to encourage a shift in human behavior. Governance systems should also be more transparent and inclusive, which will build trust between local communities and government, and provide opportunities for involvement by indigenous and local groups.

## Conclusion

Although progress has been made towards achieving the goals of the Strategic Plan for Biodiversity for the marine environment, the Aichi targets reviewed have not been fully achieved. This substantiates the predictions of GBO-4 and is unsurprising given the aspirations of the targets and the complexity of implementation. Ongoing discussions within CBD on the development of the Zero Draft of the Post-2020 Global Biodiversity Framework will set out the approach to address some of these shortfalls and provide continuing momentum towards the 2050 vision. To deliver this vision, it is important that the new targets and actions that come out of this agreed framework are fostered and implemented at a range of scales, highlighting the key need for stakeholder involvement, including local communities and youth. The UN Decade of Ocean Science for Sustainable Development (2021–2030) (“the Decade”) also provides a great opportunity to build on existing foundations to better understand changes and reverse the declines observed in our ocean. Climate change and the mainstreaming of biodiversity into policy, the economy, and society will be major challenges for marine conservation strategies and should set the context for conservation work, even—or especially—in the face of uncertainty. The rising importance of the climate change agenda and increasing recognition within sectors such as finance of the very real risk that impacts of climate change pose to the environment, economy, and community stability, could lead to more governments proactively tackling this issue. Implementing an adaptive approach to the post-2020 Framework that incorporates a range of feedback mechanisms from multiple sectors, including youth and indigenous and local communities, will be necessary to enhance marine conservation efforts while promoting economic sustainability, as well as to support the resilience of coastal communities in the face of climate change.

A common theme across the priorities identified here is the need for better recognition of global economic disparities and calls for flexible solutions in the context of socio-economic trade-offs. Critically addressing priorities to decrease overfishing, increase area-based protection, and enhance enforcement capacity—especially in cross-boundary fisheries and critical habitats—will require stakeholders to modify their perspectives on social or market-based incentives for conservation. The value of marine biodiversity varies across regions, where some nations are solely dependent on coastal waters for their citizens’ livelihoods. Appropriate measures include area-based management, restoration, and protection and fisheries recovery plans; current international fisheries should also consider economic constraints in regions projected to be less capable of tackling climate or other anthropogenic stressors. Future decision-making processes should include bioeconomics to assess capacity and the feasibility of implementing conservation strategies.

Another common theme is the need for improved sharing of data, information, and experiences among scientists, stakeholders, the public, and policy makers and improving these connections. Having sufficient baseline data and scientific understanding of marine ecosystems is essential for successful conservation efforts; therefore, the collection of useful data using a range of mechanisms (including citizen science), and data in areas with less coverage is also paramount. The knowledge and views of the public should be more readily and consistently considered in management, and novel approaches for gaining this intelligence/human capital explored. Collecting and synthesizing multiple data types using a systematic approach will be imperative. Sharing of scientific, local, and traditional knowledge coupled with equitable funding mechanisms will enable less developed countries lacking resources to implement conservation measures in their waters. Better communication, sharing experiences, and exchanging knowledge among groups, sectors, and nations will be key and should be fostered in part by encouraging accountability across these different networks.

As discussions progress on the legally binding global treaty on the conservation and sustainable use of ABNJ, there is a huge potential for a significant win for marine biodiversity and humanity alike. Coupled with the new 2050 Vision for Biodiversity; the Decade; the UNFCCC COP 26 and upcoming revisions to nations’ nationally determined contributions to addressing climate change; and the SDGs, this would offer a much brighter outlook for the future of marine biodiversity.

With the stark challenges facing global biodiversity recently highlighted by experts with regards to climate change (IPCC, 2018) and unprecedented biodiversity loss (IPBES, 2019) it is imperative now more than ever that actions are taken to implement conservation strategies in the most effective ways possible to optimize the chances of their success and of making a positive impact on the health of our marine ecosystems. We hope that focusing efforts on the priorities set out here at all scales and across all sectors will facilitate achieving this goal.

## Additional Information and Declarations

### Data Availability

The following information was supplied regarding data availability: There is no raw data associated with this review article.
